# Inspissated bile syndrome in an infant with citrin deficiency and congenital anomalies of the biliary tract and esophagus: Identification and pathogenicity analysis of a novel *SLC25A13* mutation with incomplete penetrance

**DOI:** 10.3892/ijmm.2014.1929

**Published:** 2014-09-10

**Authors:** HAN-SHI ZENG, SHU-TAO ZHAO, MEI DENG, ZHAN-HUI ZHANG, XIANG-RAN CAI, FENG-PING CHEN, YUAN-ZONG SONG

**Affiliations:** 1 Department of Pediatrics, The First Affiliated Hospital, Jinan University, Guangzhou, Guangdong 510630, P.R. China; 2 Central Laboratory, The First Affiliated Hospital, Jinan University, Guangzhou, Guangdong 510630, P.R. China; 3 Medical Imaging Center, The First Affiliated Hospital, Jinan University, Guangzhou, Guangdong 510630, P.R. China; 4 Department of Laboratory Science, The First Affiliated Hospital, Jinan University, Guangzhou, Guangdong 510630, P.R. China

**Keywords:** citrin deficiency, inspissated bile syndrome, esophageal atresia, functional analysis

## Abstract

Biallelic mutations of the *SLC25A13* gene result in citrin deficiency (CD) in humans. Neonatal intrahepatic cholestasis caused by citrin deficiency (NICCD) is the major CD phenotype in pediatrics; however, knowledge on its genotypic and phenotypic characteristics remains limited. The present study aimed to explore novel molecular and clinical characteristics of CD. An infant suspected to have NICCD as well as her parents were enrolled as the research subjects. *SLC25A13* mutations were investigated using various methods, including cDNA cloning and sequencing. The pathogenicity of a novel mutation was analyzed bioinformatically and functionally with a yeast model. Both the infant and her father were heterozygous for c.2T>C and c.790G>A, while the mother was only a c.2T>C carrier. The novel c.790G>A mutation proved bioinformatically and functionally pathogenic. The infant had esophageal atresia and an accessory hepatic duct, along with bile plug formation confirmed by laparoscopic surgery. However, the father seemed to be healthy thus far. The findings of the present study enrich the genotypic and phenotypic characteristics of CD patients, and provided clinical and molecular evidence suggesting the possible non-penetrance of *SLC25A13* mutations and the likely involvement of this gene in primitive foregut development during early embryonic life.

## Introduction

The *SLC25A13* gene on chromosome 7q21.3 was cloned, whilst its protein product, CITRIN, was designated in 1999 by Kobayashi *et al* (1). This breakthrough finding opened up a new research field on citrin deficiency (CD) and laid the foundation for subsequent investigation into this autosomal recessive disorder. Subsequently, citrin was found to be the liver-type aspartate/glutamate carrier isoform 2 (AGC2) (2,3). The laboratory and clinical characteristics of this CD, whether molecular (4–12), biochemical (13,14), medical imaging (15), hepatohistological (16–18), metabolomic (19,20), behavioral (21), therapeutic (22–26) or epidemiological (27–31), have been increasingly depicted, while patients with CD have been diagnosed not only in Asia (32–42), but also in Europe (43–46) and North America (47–49). Currently, CD has developed into a worldwide panethnic disease entity encompassing at least three age-dependent clinical phenotypes, i.e. neonatal intrahepatic cholestasis caused by citrin deficiency (NICCD) in neonates or infants, adult-onset type 2 citrullinemia (CTLN2) in adolescents and adults, as well as failure to thrive and dyslipidemia caused by citrin deficiency (FTTDCD), which was recently suggested to be a novel CD phenotype between NICCD and CTLN2 (7,11,12,19,50,51).

Although considerable laboratory and clinical progress has been made in CD research, the genotypic and phenotypic characteristics of this disease entity remain far from being completely clarified. To date, a total of 84 deleterious mutations of the *SLC25A13* gene have been reported (11,12,30,52–54), constituting valuable molecular evidence for the definite diagnosis of patients with CD. However, the pathogenicity of the majority of missense mutations was based on conventional bioinformatics evidence and studies on their direct functional effects are rather limited (10,12). Moreover, all phenotypic, therapeutic and prognostic knowledge on patients with CD has been gained through the clinical management of such patients, both pediatric and adult, and knowledge on the effects of *SLC25A13* mutations on CD fetuses remains limited (55), constituting a novel issue of perinatal medicine. In the present study, an infant with NICCD was diagnosed, who harbored a novel deleterious *SLC25A13* mutation and demonstrated inspissated bile syndrome (IBS) along with multiple congenital anomalies of the digestive system. We herein report the molecular and clinical characteristics of this case of NICCD.

## Subjects and methods

### Subjects and ethics

The research subjects in the present study were a female infant (C0218) suspected to have NICCD and her parents. The clinical findings of this family were described as a case report. The majority of the data were collected at our clinical practice or from the laboratory and imaging databases at our own hospital, with partial biochemical or imaging results from the medical records in another hospital, which were provided by the parents of the patient at the time of her referral.

This study was carried out after written informed consent was obtained from the parents of the infant prior to their enrollment in the present study. For screening analysis of the novel mutation, 60 used blood samples (with 120 *SLC25A13* alleles) for health examinations were collected as the controls. This study was approved by the Committee for Medical Ethics, the First Affiliated Hospital, Jinan University, Guangzhou, China, adhering to the World Medical Association Declaration of Helsinki (WMADH 2008), which was adopted by the 59th WMA General Assembly, Seoul, Korea, in October 2008.

### Molecular diagnosis of CD

Genomic DNA was extracted from peripheral blood samples collected from the subjects. Four high-frequency mutations of the *SLC25A13* gene, i.e., c.851_854delGTAT, c.1638_1660dup, c.615+5G>A and IVS16ins3kb, were screened by PCR/long and accurate (LA)-PCR and PCR-restriction fragment length polymorphism (RFLP) analysis. Subsequently, all 18 exons and their flanking genomic sequences were amplified by PCR/LA-PCR, and the amplified products were then sequenced, as described in our previous studies (7–9,11,15).

### RT-PCR, cDNA cloning and sequencing

As previously described by our group (9,11), EDTA anticoagulant peripheral blood samples were collected, the lymphocytes were isolated with lymphocyte separation medium (LSM, MP) and then homogenized immediately in RNAiso Plus (Takara Bio Inc., Otsu, Japan) to extract the total mRNA following the manufacturer’s instructions. Subsequently, the *SLC25A13* transcripts were reverse-transcribed and amplified by PCR, and the purified products were cloned into the pSIMPLE-18 *Eco*RV/BAP Vector (Takara Bio Inc.) and transformed into DH5α *Escherichia coli* competent cells. To examine the co-segregation of the 2 *SLC25A13* variations detected in the family, only the cDNA clones containing exons 1 and 8 together were selected for further sequencing analysis, since the 2 variations occurred in these 2 exons, respectively. The sequencing results of the cDNA clones were aligned with the *SLC25A13* mRNA sequence to judge the parental origins of the 2 variations.

### Screening of the novel missense variation in controls

A nested PCR-RFLP procedure was performed in the present study for the screening for the novel missense variation in the control individuals. The nucleotide sequences of the forward and reverse primers were as follows: 5′-TCACTCATTCCAGT GCCTTG-3′ (IVS6F) and 5′-CAATGCCGCAAAGGCAA CTG-3′ (IVS8B) in the first PCR; and 5′-GAGTTTGTTC TGGCAGCACAG-3′ (Ex8F) and 5′-TATTTCAGTATAG CCTTCAGTTTGG-3′ (Ex8R) in the second. The temperature profile was 94°C for 5 min followed by 40 cycles (20 cycles in the second PCR) of 94°C for 30 sec, 59°C for 40 sec and 72°C for 1.0 min, and a final extension step at 72°C for 10 min. The restriction endonuclease for RFLP analysis was Hpy188I (New England Biolabs Inc., Ipswich, MA, USA). For frequency calculation of the variation, the mutated allele number was divided by the total allele number of the *SLC25A13* gene in all controls and then the quotient was amplified by 100%.

### Bioinformatics analysis

The conservative property of the amino acid affected by the novel missense mutation was surveyed as described in our previous publication (11). Briefly, using a comparative alignment software of Genetyx^®^ version 7.1 (Genetyx Co., Tokyo, Japan), the amino acid sequences of human citrin and aralar were aligned with those in the homologous proteins from 10 different eukaryotic species, including chimpanzee, dog, mouse, rat, chicken, *Xenopus tropicalis*, macaque, *Caenorhabditis elegans*, opossum and cow. The amino acid sequences of the homologous proteins were collected from ENSEMBL at http://www.ensembl.org/index.html. Moreover, 2 online tools, MutationTaster (http://mutationtaster.org/MutationTaster/index.html) and PolyPhen-2 (http://genetics.bwh.harvard.edu/pph2/), were used in this study to evaluate the pathogenic potential of the novel missense mutation. In the first software, a probability value close to 1 indicates a high ‘security’ of the prediction and in the second software used, the mutation with a probabilistic score >0.85 is classified as ‘probably damaging’, while a score >0.15 is classified as ‘possibly damaging’, as recently described (12).

### Functional effects of the novel missense mutation

A yeast model with a disruption of the *agc1* gene, which is highly homologous to human *SLC25A13*, was applied in the present study to evaluate the functional effects of the novel missense mutation. The diploid *agc1*-disrupted yeast strain, BY*agc1Δ*, was the same as the one used in our previous publication (12). The normal citrin-encoding cDNA sequence was amplified and recombined into the expression vector, pYX212 (Novagen Inc., Madison, WI, USA), to form the plasmid, pYX212-citrin. The novel missense mutation was introduced into the wild-type cDNA by overlap-extension PCR, and the generated variants were cloned into pYX212 to constitute the plasmid pYX212-mutant. Subsequently, transformation of the BY*agc1Δ* strain with the empty plasmid pYX212 (vector), pYX212-mutant and pYX212-citrin control (citrin) was carried out, and the transformed strains were cultured in SA medium with acetate as the unique carbon source. The growth abilities of the transformed strains were examined after 96 h of culture by measuring the OD_600_, and the data were analyzed with one-way ANOVA followed by the Bonferroni method to compare the differences in the mean values among the different groups, with a value of P<0.05 considered to indicate a statistically significant difference. All raw data were logarithmically transformed in the case of non-homogeneity of variance prior to statistical comparison.

## Results

### Case report

**Figure 1:**
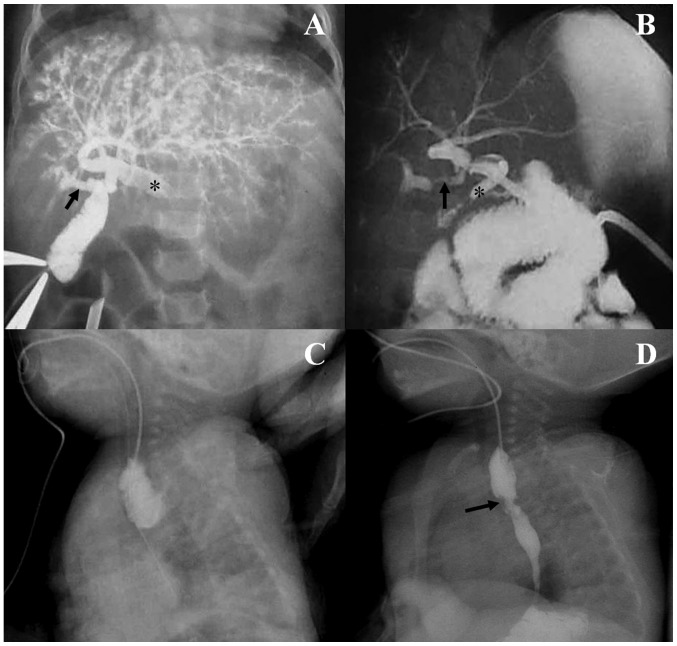
Cholangiography via punctured gallbladder and esophageal contrast radiography. Note the filling defect in the common bile duct (asterisk) and the dilated upstream biliary tract without contrast flow into the duodenum, and the accessory right hepatic duct (arrow) joining the cystic duct (A). A postoperative contrast study (B) via the T-tube placed into the common bile duct (asterisk) displayed a right bile duct stricture (arrow) where the gallbladder had been surgically removed. Note the free contrast passage into the duodenum. Esophageal contrast radiography (C) before gastroesophagostomy showed obvious dilatation of the upper esophagus, without contrast flow into the trachea, lower esophagus or stomach, while postoperative contrast radiography (D) displayed the contrast flow through a mild annular stricture (arrow) between the upper and middle esophagus.

A 7-month-old female infant was referred to our hospital due to prolonged jaundice for approximately 4 months and growth retardation for 1.5 months. Her jaundice (yellow skin and sclera) drew the attention of her parents at the age of 3.3 months. A liver function test at the local hospital revealed elevated cholestatic indices, including gamma-glutamyl transpeptidase (GGT), direct bilirubin (Dbil) and total bile acid (TBA) (Table I). Due to the prolonged jaundice and unresolved laboratory abnormalities, the infant was referred to another hospital at the age of 4 months, where magnetic resonance cholangiopancreatography revealed the dilatation of common hepatic and bile ducts. Laparoscopy was thus performed when the infant was aged 4.5 months, and an intraoperative cholangiography displayed biliary tree dilatation and filling defect in the common bile duct, along with an accessory hepatic duct (AHD) joining the cystic duct (Fig. 1A). A cholecystectomy was subsequently performed, an intrabiliary thick plug in the color of dark green was removed, and bile duct irrigation and choledochostomy with T-tube drainage was carried out. Pathological analysis of the plug confirmed the diagnosis of inspissated bile syndrome (IBS). Thereafter, the infant’s jaundice subsided and the laboratory indices gradually improved (Table I); the infant was discharged at the age of 5 months. Half a month later, nevertheless, a physical examination revealed that her body weight was 5.34 kg (−3.6 SD), her length was 59 cm (−3.2 SD) and her head circumference was 39 cm (−2.5 SD). Another anthropometric test at the age of 6 months revealed a weight of 6.0 kg (−2.5 SD) and a length of 62.0 cm (−2.2 SD). The growth retardation continued in the following month, and the infant was referred to our hospital at the age of 7 months for further evaluation, following the removal of the drainage T-tube.

The infant was born to a non-consanguineous couple after 37 weeks of uneventful gestation with a birth weight of 2.25 kg. On the first day after birth, the infant was admitted to hospital due to vomiting and respiratory distress. Contrast imaging of the upper digestive tract revealed the existence of esophageal atresia (EA) (Fig. 1C), which was then resolved by a gastroesophagostomy under general anesthesia. The couple had experienced 2 pregnancies prior to this one, but both were aborted in the first trimester. Both parents appeared healthy, without any clinical symptoms or signs of CTLN2. There was no known family history of any genetic disease.

Physical examination at referral revealed a weight of 6.25 kg (−2.6 SD), a length of 63.0 cm (−3.3 SD) and a head circumference of 42 cm (−1.0 SD). There was no dysmorphic appearance, only a slightly chubby face. There was no evidence of jaundice (yellow skin and sclera) and there were no visible petechiae or ecchymoses. No pallor or cyanosis of the lips were observed. There was no tachypnea or dyspnea, and no stridor, wheezes, crackles or crepitus could be heard on auscultation of the both lungs. The heart sounds were normal without audible murmurs or arrhythmia. Upon abdominal inspection, no dilated veins or abdominal distention were observed. The liver was palpated with a soft edge 2 cm below the right costal margin in the mid-clavicular line. Her spleen was not palpable. A neurological examination revealed slightly reduced muscle tone. There was no neck stiffness, knee reflex was normal and there was no positivity for Brudzinski’s or Kernig’s sign.

Following biochemical analysis, no abnormal liver function index was observed, although the serum GGT level was elevated, suggesting the existence of cholestasis, as shown in Table I. Taking into consideration her prolonged jaundice, growth retardation and chubby face, and the 2 first-trimester miscarriages of her mother, *SLC25A13* gene analysis was performed on the family to evaluate the possibility of a diagnosis of CD. A lactose-free and MCT-enriched therapeutic formula was subsequently introduced, while a diet rich in protein and lipids was also encouraged. A clinical following-up 4 months later revealed that the weight of the infant was 7.9 kg (−1.6 SD), her length was 70 cm (−1.5 SD) and her head circumference was 44 cm (−0.8 SD), along with a marked improvement (complete recovery) of the cholestatic indices, GGT, TBA and Dbil (Table I).

### SLC25A13 genotypic characteristics of the family

**Figure 2:**
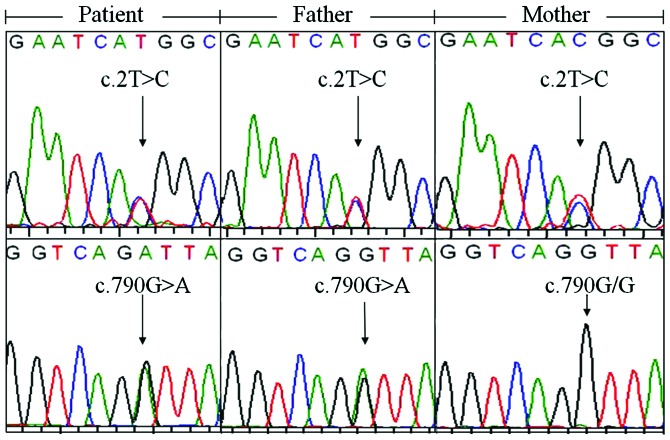
*SLC25A13* gene variations in the family unveiled by direct DNA sequencing. The patient and her father both harbored the c.2T>C and c.790G>A (p.V264I) variations, while the mother was only a carrier of the former variation.

High-frequency mutation screening did not reveal any *SLC25A13* mutation. However, direct DNA sequencing revealed that both the infant and her father harbored c.2T>C and c.790G>A (p.V264I) variations, while the mother was only a carrier of c.2T>C (Fig. 2). To the best of our knowledge, c.790G>A is a novel *SLC25A13* variation that has not been previously reported. Following *SLC25A13* cDNA cloning analysis, from a total of 27 clones from the infant, 7 were found to harbor c.2T>C, another 18 carried c.790G>A, 1 had neither, and the remaining clone had both variations. Analysis of the cDNA clones from the father revealed similar characteristics. The total 29 cDNA clones consisted of 9 clones with c.2T>C, 13 with c.790G>A, 3 with neither, and 4 with both variations. These findings clearly indicated that the 2 variations were both biallelic, not only in the infant displaying clinical characteristics, but also in her father who did not show any symptoms or signs of CD to date.

### Bioinformatic evidence of the pathogenicity of the novel mutation

**Figure 3:**
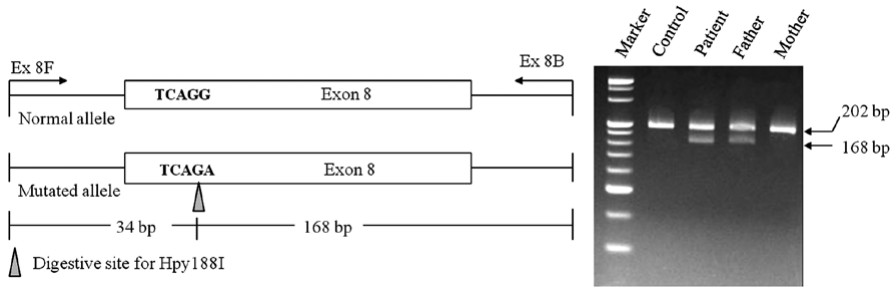
Nested PCR-RFLP protocol for the screening of the c.790G>A(p.V264I) variation. (Left panel) Schematic diagram of the enzymatic digestive protocol. The variation generated a digestive site for the enzyme Hpy188I, producing 2 fragments of 34 and 168 bp. PCR amplification of the normal allele gave rise to an expected product of 202 bp. (Right panel) Representative gel electrophoresis for the PCR products digested with Hpy188I, showing a band of 202 bp from the normal allele, and another band of 168 bp due to the variation. Note that the patient and her father were both heterozygous for the variation, while the mother was not.

**Figure 4:**
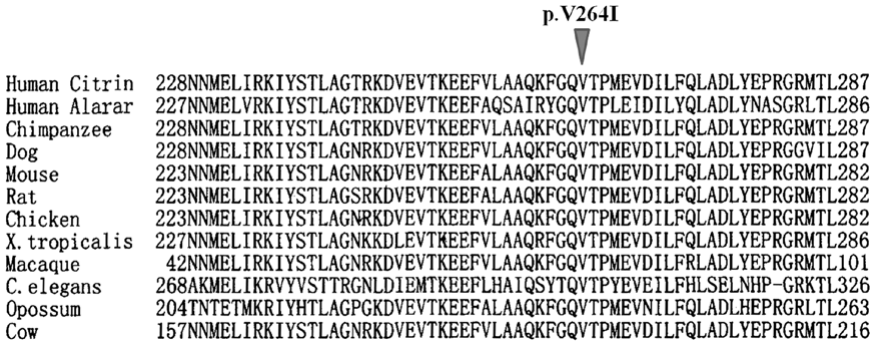
Comparative alignment of the amino acid sequences of the homologous proteins. The mutation p.V264I affected an amino acid highly conservative in all the 12 homologous proteins from 11 eukaryotic species including human, chimpanzee, dog, mouse, rat, chicken, *Xenopus tropicalis*, macaque, *Caenorhabditis elegans*, opossum and cow.

Using the newly-developed nest PCR-RFLP protocol (Fig. 3), c.790G>A (p.V264I) was screened in 60 control samples and no carrier was found, indicating a frequency of <1%. The comparative alignment of the amino acid sequences of the homologous protein in a diversity of species (Fig. 4) documented the highly conservative property of the valine at codon 264 affected by this mutation. The probability value of >0.9999 upon MutationTaster analysis strongly indicated its deleterious nature, but according to the results produced by Polyphen-2 analysis, this mutation was predicted to be benign with a score as low as 0.001. This discrepancy necessitated conducting functional analysis for this novel missense mutation.

### Effect of the novel mutation on the AGC2 function of citrin protein

**Figure 5:**
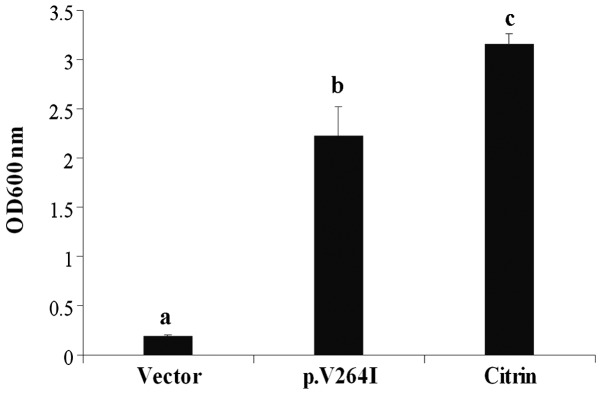
Effect of the novel c.790G>A(p.V264I) mutation on the AGC2 function of citrin protein. The *agc1Δ* yeast strains transformed with empty plasmid pYX212 (vector), pYX212-mutant (p.V264I) and pYX212-CITRIN control (citrin) were cultured in SA medium for 96 h, and their growth abilities were examined by measuring OD_600 nm_. The results are the means ± SD of 6 repeated experiments, and different letters above the bars indicated a statistically significant difference (P<0.01).

As illustrated in Fig. 5, the growth ability of the BY*agc1Δ* yeast transformed with the mutant plasmid (p.V264I) was significantly reduced (P<0.01) in comparison with that transformed with the plasmid pYX212-citrin (citrin). However, when compared with the empty plasmid group (vector), the growth ability in the mutant group (p.V264I) was still higher (P<0.01). These findings indicated that the p.V264I mutation reduced, but did not eliminate the AGC2 function of citrin protein.

## Discussion

In previous studies, the c.2T>C variation frequency was found to be 3.0% (6/200) in a Chinese (9), and approximately 2.8% (85/3074) in a Thai population (31), both suggesting that it may be a single-nucleotide polymorphism (SNP) of the *SLC25A13* gene. This initiation codon variation gave rise to a citrin molecule lacking the first 34 amino acid residues, and this truncated protein lost the ability to localize within the mitochondrial membrane, thereby leading to an almost complete loss of AGC2 function (10). Although the frequency of this deleterious SNP has been proven to be rather high, a homozygous variation (a patient with CD) has yet to be identified. A possible explanation for this issue is the homozygote lethality. In the present study, the two spontaneous miscarriages of the mother in the first trimester may reflect the high pathogenicity of this SNP. It was very likely that the two aborted fetuses (possibly both with homozygous SNP) could not survive through an uneventful pregnancy. As regards the c.790G>A mutation, in addition to the bioinformatic evidence supporting its disease-causing characteristic, the functional analysis in this study provided direct eukaryotic evidence further solidifying its pathogenicity. However, this missense mutation caused the reduction, but not the elimination of AGC2 function of citrin protein, as illustrated in Fig. 5. This is the most likely explanation for the survival of this infant through the entire pregnancy, and her delivery as a full term baby. This novel c.790G>A mutation, along with the c.2T>C variation, constituted reliable diagnostic evidence for NICCD in the infant, and further expanded the mutation spectrum of the *SLC25A13* gene.

The term IBS, also known as bile plug syndrome, was used to indicate patients with prolonged jaundice in whom normal extrahepatic bile ducts containing inspissated bile were found at surgery for presumed extrahepatic biliary atresia (56). Although cholestasis has been reported as a characteristic histological characteristic of NICCD (17), such a large bile plug causing obstruction of the common bile duct as in this infant has not been reported previously in patients with NICCD. The contributing factors for IBS included Rh and ABO incompatibility, blood transfusion, parenteral nutrition in pre-term infants, diuretic medication, bowel dysfunction and disseminated intravascular coagulation (57). None of these factors was found in this infant, and CD may be a very likely contributing etiology for her IBS. Actually, canalicular bile secretion involves a series of ATP-binding cassette transporters as export pumps for bile salts and other organic solutes (58), and among these, the bile salt export pump (BSEP), a pump noteworthy in particular, transports bile acids across the apical membrane and constitutes the major determinant and driving force for the generation of bile flow (59). Since CD causes energy shortage in the liver (12,24), the function of these transporters, including BSEP, would thereby be affected inevitably in this infant with NICCD, causing deficit of the major driving force, disturbing her generation of bile flow, giving rise to intra- and extrahepatic cholestasis, and finally resulting in IBS formation.

Accessory bile ducts are rare congenital anomalies of the primitive foregut bud during the development of the biliary tract before 5 weeks of gestational age; apart from their importance to the radiologist and to the biliary and hepatic transplant surgeon, these anomalies may be associated with congenital lesions elsewhere (60). In the present study, the infant with NICCD with AHD also suffered from EA, another malformation originated from the division of the primitive foregut into ventral respiratory and dorsal esophageal parts during the 4th week of embryonic life (61). Although the underlying mechanisms remain unknown, EA has been recognized a multifactorial complex disease with the involvement of genetic and environmental factors, and has been reported to be associated with some single-gene disorders, such as Feingold syndrome, CHARGE, anophthalmia-oesophageal-genital (AEG) and Opitz G syndrome (61,62). To the best of our knowledge, congenital anomalies of the digestive system in patients with CD have been rarely reported, although a Chinese infant with NICCD with congenital biliary atresia, confirmed by abdominal laparoscopy and liver biopsy, was previoulsy reported (54). In the present study, we reported the concurrent existence of AHD and EA in an infant with NICCD. These findings suggest that the *SLC25A13* mutation may be an additional contributing genetic factor leading to congenital anomalies of the primitive foregut during the early stage of fetal development, although more molecular, embryonic and histological evidence is required in order to address this issue.

Non-penetrance refers to the lack of clinical signs and symptoms in genetically affected individuals. This phenomenon is not uncommon, not only in Mendelian disorders inherited as autosomal dominant traits (63,64), but also in some autosomal recessive diseases, such as Brown-Vialetto-Van Laere syndrome (65) and Wolfram syndrome (66). Whether or not CD penetrance is complete has remained an unresolved issue for years (27). However, there have been several reports on adult individuals harboring the biallelic but non-penetrant mutations of the *SLC25A13* gene, at least at the age these individuals were when these mutations were reported. Two adult siblings had been definitely diagnosed as being homozygous for the c.851_854del4 mutation, one demonstrating the typical clinical and laboratory manifestations of CTLN2, while the other did not (67). In addition, a girl with the *SLC25A13* genotype c.851_854del4/c.1799_1800insA displayed characteristics of NICCD, but her mother heterozygous for the mutations c.1799_1800insA and IVS16ins3kb did not display any symptoms of CTLN2, as it recently demonstrated by a Japanese group (68). In the present study, we reported similar findings. The lack of CTLN2 phenotypic characteristics in the father, who had the same genotype as that of the infant with NICCD, lent further support to the concept that CD penetrance may be incomplete, and suggested the existence of other environmental, genetic or epigenetic factors that may modulate the onset of CTLN2.

In conclusion, in this study, we reported two individuals in the same family both harboring the same biallelic variations, but demonstrating markedly different phenotypic features. The infant had IBS and multiple anomalies of the digestive system, while the father appeared healthy. Bioinformatically and functionally, the c.790G>A variation proved to be a novel pathogenic mutation of the *SLC25A13* gene. These findings further enrich the clinical and molecular spectrum of NICCD, and suggest the existence of CD non-penetrance and the possible involvement of *SLC25A13* in primitive foregut development during early embryonic life.

## Figures and Tables

**Table I:** Dynamic alterations of the biochemical indices in the infant with citrin deficiency.

Biochemical indices	3.3 M	3.5 M	4.0 M	4.3 M	4.5 M^a^	4.6 M	4.7 M	4.8 M	5.0 M	5.5 M	5.8 M	7.0 M^b^	11 M
ALT (5–40 U/l)	49	44	100	138	198	99	137	83	58	40	55	36	27
AST (5–40 U/l)	66	58	88	118	124	75	113	62	50	37	67	37	45
GGT (8–50 U/l)	444	411	537	532	388	313	465	508	-	364	134	318	34
ALP (20–500 U/l)	618	588	1133	1209	1332	1011	1428	944	643	174	426	420	399
TP (60.0–83.0 g/l)	56.9	54.9	61.2	56.1	50.0	56.8	60.8	68.5	73.5	-	61.6	68.7	68.6
Alb (35.0–55.0 g/l)	40.2	39.7	41.7	39.4	34.6	40.5	43.5	45.4	49.0	-	45.5	50.0	51.1
Glb (20.0–30.0 g/l)	16.7	15.2	19.5	16.7	15.4	16.3	17.3	23.1	24.5	-	16.1	18.7	17.5
Tbil (2–19 μmol/l)	67.7	59.2	123.6	149.8	204.5	148.3	135.9	67.3	45.4	20.3	13.2	3.9	5.5
Dbil (0–6 μmol/l)	53.8	48.7	96.3	118.4	161.6	120.1	110.8	57.4	40.1	13.0	9.4	1.8	1.4
Ibil (2.56–20.9 μmol/l)	13.9	10.5	27.3	31.4	42.9	28.2	25.1	9.9	5.3	7.3	3.8	2.1	4.1
TBA (0–10 μmol/l)	157.5	152.8	135.1	112.3	173.3	26.6	26.9	5.5	4.7	49.33	8.7	7.3	2.1

M, months (age of infant).

a Time at which laparoscopic surgery was performed, and

b time of referral to our hospital.

ALT, alanine transaminase; AST, aspartate transaminase; GGT, gamma-glutamyl transpeptidase; ALP, alkaline phosphatase; TP, total protein; Alb, albumin; Glb, globulin; Tbil, total bilirubin; Dbil, direct bilirubin; Ibil, indirect bilirubin; TBA, total bile acid.

## Abbreviations

CD: citrin deficiency
NICCD: neonatal intrahepatic cholestasis caused by citrin deficiency
CTLN2: adult-onset type 2 citrullinemia
FTTDCD: failure to thrive and dyslipidemia caused by citrin deficiency
AGC2: aspartate/glutamate carrier isoform 2
GGT: gamma-glutamyl transpeptidase
Dbil: direct bilirubin
TBA: total bile acid
